# In Vivo Imaging of mGluR5 Changes during Epileptogenesis Using [^11^C]ABP688 PET in Pilocarpine-Induced Epilepsy Rat Model

**DOI:** 10.1371/journal.pone.0092765

**Published:** 2014-03-24

**Authors:** Hongyoon Choi, Yu Kyeong Kim, So Won Oh, Hyung-Jun Im, Do Won Hwang, Hyejin Kang, Boeun Lee, Yun-Sang Lee, Jae Min Jeong, E. Edmund Kim, June-Key Chung, Dong Soo Lee

**Affiliations:** 1 Department of Nuclear Medicine, Seoul National University College of Medicine, Seoul, Republic of Korea; 2 Department of Nuclear Medicine, Seoul National University Boramae Medical Center, Seoul, Republic of Korea; 3 Institute of Radiation Medicine, Medical Research Center, Seoul National University, Seoul, Republic of Korea; 4 Department of Molecular Medicine and Biopharmaceutical Science, Graduate School of Convergence Science and Technology, Seoul National University, Seoul, Republic of Korea; Kaohsiung Chang Gung Memorial Hospital, Taiwan

## Abstract

**Introduction:**

Metabotropic glutamate receptor 5 (mGluR5) that regulates glutamatergic neurotransmission contributes to pathophysiology of epilepsy. In this study, we monitored the changes of mGluR5 *in vivo* using [^11^C]ABP688 PET during the epileptogenesis in a pilocarpine-induced epilepsy rat model.

**Methods:**

*In vivo* mGluR5 images were acquired using [^11^C]ABP688 microPET/CT in pilocarpine-induced chronic epilepsy rat models and controls. We also acquired microPET/CT at acute, subacute as well as chronic periods after status epilepticus. Non-displaceable binding potential (BP_ND_) of [^11^C]ABP688 was calculated using simplified reference tissue model in a voxel-based manner. mGluR5 BP_ND_ of the rat brains of epilepsy models and controls were compared.

**Results:**

Status epilepticus developed after pilocarpine administration and was followed by recurrent spontaneous seizures for more than 3 weeks. In chronic epilepsy rat model, BP_ND_ in hippocampus and amygdala was reduced on a voxel-based analysis. Temporal changes of mGluR5 BP_ND_ was also found. In acute period after status epilepticus, mGluR5 BP_ND_ was reduced in the whole brain. BP_ND_ of caudate-putamen was restored in subacute period, while BP_ND_ of the rest of the brain was still lower. In chronic period, global BP_ND_ was normalized except in hippocampus and amygdala.

**Conclusions:**

*In vivo* imaging of mGluR5 using [^11^C]ABP688 microPET/CT could successfully reveal the regional changes of mGluR5 binding potential of the rat brain in a pilocarpine-induced epilepsy model. The temporal and spatial changes in mGluR5 availability suggest [^11^C]ABP688 PET imaging in epilepsy provide abnormal glutamatergic network during epileptogenesis.

## Introduction

Glutamate mediated neurotransmission is important in the pathogenesis of epilepsy. Metabotropic glutamate receptors (mGluRs) play a role in the initiation of epileptic discharge and propagation [1]–[3]. In particular, group I mGluRs (mGluR1 and mGluR5) are involved in making abnormal synaptic plasticity which induce long-lasting depolarization and activate neurons to persistently hyperexcitable state [3]–[5]. Therefore, there has been a growing interest in mGluR-mediated neuronal transformation to develop spontaneous recurrent seizures, which contributes crucially to epileptogenesis.

The abnormalities of mGluR expression in epilepsy were found both in human and animal studies. In focal cortical dysplasia, strong immunoreactivity of group I mGluRs in dysplastic neuronal cells suggested possible contribution of mGluRs to epileptogenesis [6]. In human temporal lobe epilepsy (TLE), Blümcke, et al. reported up-regulated mGluR1 though mGluR5 did not show any significant change [7], however, Notenboom, et al. showed up-regulation of mGluR5 in TLE patients, particularly in hippocampal non-sclerosis groups [8]. In pilocarpine-induced epilepsy animal models, mGluR5 protein expression decreased in the hippocampus, and mGluR-mediated hippocampal long term depression (LTD) was reduced [9], [10]. Though mGluR expression results were inconsistent in epilepsy studies, mGluRs were proposed to be an important molecular target for developing new antiepileptic drugs [2], [11].

Recently, a positron emission tomography (PET) tracer, 3-(6-methyl-pyridin-2-ylethynyl)-cyclohex-2-enone-O-^11^C-methyl-oxime ([^11^C]ABP688), was developed as a highly selective antagonist of mGluR5 [12]. As well as in animal models, using [^11^C]ABP688 PET, mGluR5 status was examined in patients with major depression or in smokers and ex-smokers [13], [14]. Furthermore, the studies to measure mGluR5 receptor availability based on the tracer kinetics for reversible ligands were performed in humans and rodents [15], [16].

As mGluR5 is supposed to be involved in epileptogenesis, we examined the localized abnormalities of mGluR5 in a chronic epilepsy rat model using [^11^C]ABP688 PET. We also studied temporal patterns of mGluR5 availability after status epilepticus and tried to understand the abnormal glutamatergic networks in epileptogenesis using a pilocarpine-induced epilepsy model.

## Materials and Methods

### Establishment of epilepsy rat model

Twenty-two adult male Sprague-Dawley (SD) rats (7 weeks old; Koatech, Seoul, Korea), weighing 180–200 g were used as controls (n = 7) and models (n = 15). They were kept at standard laboratory condition (22–24°C, 12 hour light and dark cycle) with free access to water and standard feed. All the experimental procedures were approved by Institutional Animal Care and Use Committee at Seoul National University Hospital (IACUC Number 13-0224).

Rats were pretreated with lithium chloride (127 mg/kg, i.p., Sigma, St. Louis, MO) and methylscopolamine-bromide (1 mg/kg, i.p. Sigma) 24 hours and 30 min before pilocarpine administration, respectively. Pilocarpine hydrochloride (30 mg/kg, i.p., Sigma) was injected to trigger status epilepticus. Repeated doses of pilocarpine hydrochloride for 10 mg/kg were then administered every 30 min until stage 4 seizures developed according to the Racine scale [17]. The control group received lithium chloride, methylscopolamine-bromide and saline (sham treatment) instead of pilocarpine. Status epilepticus was defined as continuous generalized seizures with stage 4 or 5 according to Racine scale without normal behavior between seizures. Diazepam (10 mg/kg, i.p. Samjin, Seoul, Korea) was injected 60 min after the onset of status epilepticus to terminate seizure activity. Repeated diazepam (5 mg/kg) was administered unless status epilepticus was terminated to reduce mortality. After cessation of status epilepticus, rats were treated with supplementary moistened and crushed pellets soaked in Gatorade on the cage floor and given 5 mL i.p. injection of 0.9% saline for hydration in the rats unable to drink. Among 15 rats of experimental group, 11 rats survived in acute and subacute periods to yield 5 rats for PET examination in chronic period.

The model rats in chronic period were monitored using a video recorder (12 h/day, for 2 days) to evaluate spontaneous recurrent seizure. Spontaneous recurrent seizures were observed in all chronic epilepsy rats.

### PET experimental design

PET scans were acquired in chronic epilepsy rats and controls. Chronic period was more than 3 weeks after status epilepticus (median 44 d, range 34 d–59 d). In 7 control rats, PET scans were acquired median 33 d (range 18 d–52 d) after sham treatment.

**Figure 1:**
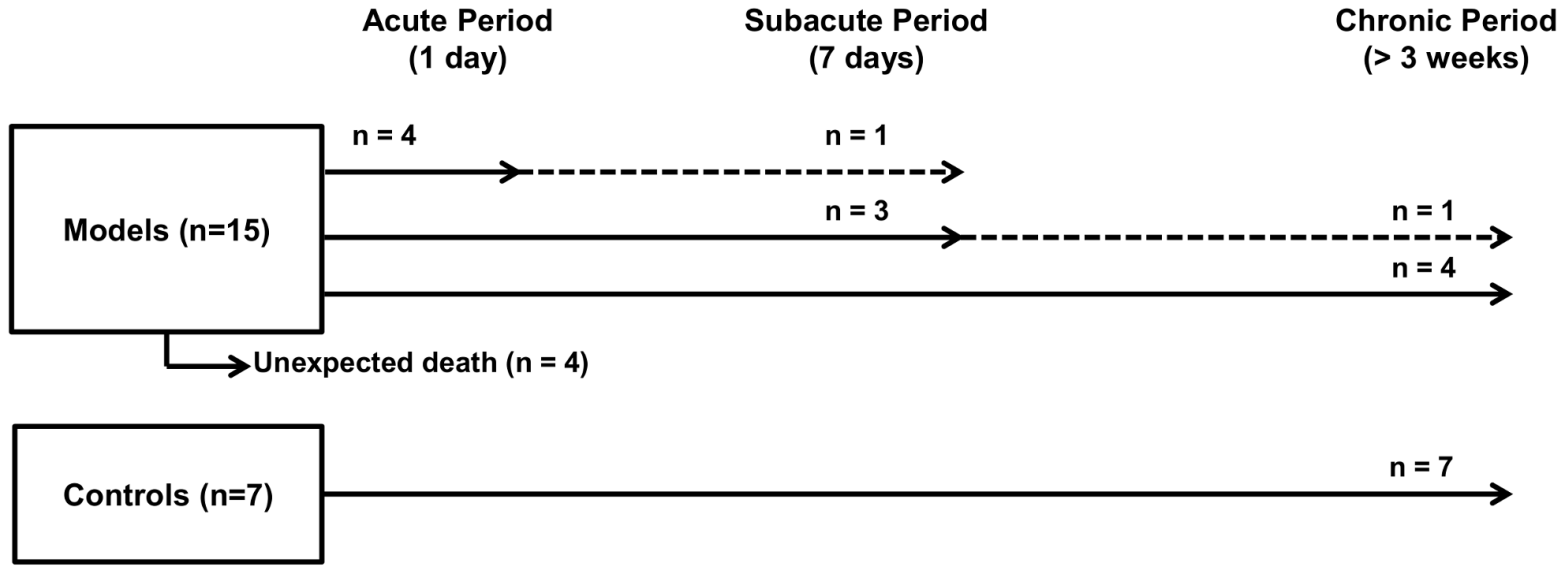
Numbers of PET scans at each time point. After status epilepticus induced by pilocarpine administration, four rats were scanned in acute period. Three rats were scanned in subacute period without PET acquisition in acute period and four rats were scanned in chronic period after status epilepticus. Among the four rats scanned in acute period, a rat was scanned in subacute period and among the three rats scanned in subacute period, a rat was scanned in chronic period, repetitively (dotted line).

To find temporal changes after status epilepticus, PET scans were acquired at acute and subacute periods in experimental group: acute period was defined as 1 day after status epilepticus, and subacute period defined as 7 days after status epilepticus. Because of general condition of rats, all the rats were not repetitively scanned for each period. PET scans were successfully obtained for 4 rats in acute period, 4 rats in subacute period and 5 rats in chronic period after status epilepticus. PET experiments in this study were summarized in Figure 1.

### Synthesis of [^11^C]ABP688

ABP688 and desmethyl-ABP688 were prepared according to previously reported method [12] and all synthesized products were confirmed by ^1^H-NMR and mass spectroscopy. All other chemical reagents were used as purchased without any purification.

[^11^C]ABP688 synthesis was performed in a closed and lead-shielded hot cell by loop method [18]. Briefly, 60% sodium hydride (5 mg, 0.12 mmol) was added to a solution of desmethyl-ABP688 (1 mg, 4.42 μmol) in anhydrous *N,N*-dimethylformamide (200 μL) and then the mixture was filtered. The resulting solution was loaded into stainless steel loop of injection port of HPLC. [^11^C]CH_3_I produced from [^11^C]CO_2_ using TRACERlab FX C Pro module (GE Medical Systems, Sweden) was passed through the loop and reacted with precursor at room temperature for 3 min. The product was purified by preparative HPLC (Xterra preparative column RP8, 10 μm, 10×250 mm, Waters; mobile phase, EtOH:10 mM NaH_2_PO_4_ [50∶50], isocratic; flow rate, 4 mL/min). The purified [^11^C]ABP688 (retention time of 10–11 min) was passed through a sterile Millex FG and collected in a sterile vial. The final product was diluted with sterile saline for injection.

The radiochemical purity and specific activity of [^11^C]ABP688 were determined by the analytical HPLC (Xterra analytical column RP18, 3.5 μm, 4.6×100 mm, Waters; mobile phase, acetonitrile∶water [50∶50], isocratic; flow rate, 1.5 mL/min). The retention time of [^11^C]ABP688 in analytical HPLC was 4–4.5 min.

### [^11^C]ABP688 PET acquisition

PET scans were performed on a dedicated microPET/CT scanner (eXplore VISTA, GE Healthcare). All animals were anesthetized and maintained with 1% isoflurane at 1 L/min oxygen flow and placed on the prone position under a scanner. Rats received an intravenous bolus injection (0.2–0.5 mL/rat) of [^11^C]ABP688 (7.0–17.1 MBq/100 g) and list-mode data were acquired for 60 min with the energy window 400–700 keV. These list mode data were framed into a dynamic sequence of 6×30 s, 7×60 s, and 5×600 s frames. The images were reconstructed by a 3-dimensional ordered-subsets expectation maximum (OSEM) algorithm with attenuation, random and scatter correction. The voxel size was 0.3875×0.3875×0.775 mm.

### Image preprocessing, kinetic modeling and parametric mapping

Individual summed PET images were obtained from all rats (0–60 min summed images) followed by manual cropping of the necessary parts to include the entire brain. For the seven control rats, images were spatially normalized to a representative brain. A voxel-based average brain image was constructed using nonlinear warping method after linear affine transformation. All transformations were performed using BioImage Suite software package (www.bioimagesuite.org, Yale University) and registration was visually confirmed. In order to obtain [^11^C]ABP688 PET template, the mean image was co-registered with a standard MRI T2 template using linear affine transformation [19]. All the PET images including experimental and control groups were spatially normalized to the [^11^C]ABP688 PET template.

Regional time-activity curves (TACs) were calculated using the pre-defined volumes of interest (VOIs) on the rat template consisting of caudate-putamen, hippocampus, amygdala, frontal cortex, and cerebellum [19]. For quantitative analysis, we used kinetic modeling analysis and generated parametric images of [^11^C]ABP688 PET. Non-displaceable binding potential (BP_ND_) was used to evaluate receptor availability, BP_ND_ was calculated by the simplified reference tissue model (SRTM) [20], [21] and the cerebellum was used as a reference region for the mGluR5 quantification [16].

Kinetic analyses and voxel-based BP_ND_ mapping were performed using MATLAB (MathWorks, Natick, MA) and C-based programs of Turku PET center (http://www.turkupetcentre.fi, Turku PET Centre, Finland). Parametric BP_ND_ maps were smoothened with a Gaussian filter of 1.2 mm full width at half maximum (FWHM).

### Voxelwise analysis of parametric maps

The BP_ND_ parametric maps of the rats in chronic period were compared with those of control rats on a voxel basis. Two-sample t-test was performed between two groups using statistical parametric mapping software package (SPM2; University College London, London, England). Uncorrected values of P<0.001 were set as the significance threshold and an extent threshold of 30 contiguous voxels was applied.

In order to evaluate the significance of changes in BP_ND_ of chronic epilepsy models, and to re-confirm the results of the voxel-based analysis, a post-hoc VOI analysis was performed using nonparametric Mann-Whitney test in the significant clusters with decreased BP_ND_.

### Temporal changes of mGluR5 BP_ND_

We obtained mGluR5 BP_ND_ of predefined VOIs including caudate-putamen, hippocampus and amygdala in models in different periods and controls. Using spatially normalized BP_ND_ maps and the VOIs, regional BP_ND_ was obtained in models and controls. Regional BP_ND_ in epilepsy models in acute, subacute, and chronic periods were compared with that in controls.

### Statistical analysis

Data were expressed as mean ± SD. To test for differences in the regional BP_ND_ obtained from predefined VOIs, nonparametric Mann-Whitney test was performed between models in different periods and controls. We also performed Mann-Whitney test in post-hoc voxel-based analysis, i.e. comparison of BP_ND_ in the significant clusters between models in chronic period and controls. The statistical analyses were performed using SPSS software (version 18; SPSS Inc., Chicago, IL).

## Results

### Time-activity curve for [^11^C]ABP688

**Figure 2:**
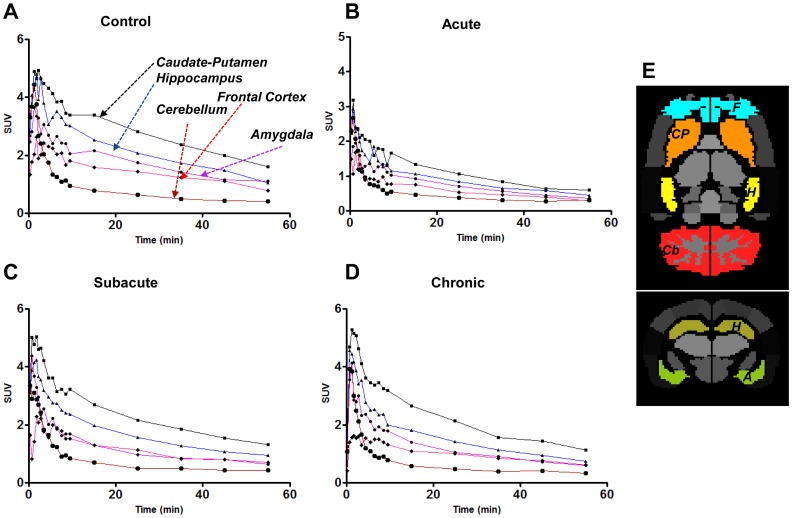
Representative time activity curves (TACs) of experimental epilepsy models. TACs in the caudate, hippocampus, frontal cortex, amygdala and cerebellum for 60[^11^C]ABP688 PET in the control (A), and epilepsy model rats in acute period (B), subacute period (C), and chronic period (D) after pilocarpine-induced status epilepticus. (E) Predefined VOIs for TACs and quantitative analyses. CP: Caudate-putamen; H: Hippocampus; F: Frontal cortex; Cb: Cerebellum, and A: Amygdala. SUV: Standardized uptake unit, defined as activity (MBq/mL) within the volume of interest divided by the injected dose per body weight (MBq/g).

Figure 2 shows representative time-activity curves (TACs) of controls and models in acute, subacute, and chronic periods. TACs were drawn for 1) caudate-putamen, 2) hippocampus, 3) frontal cortex, 4) amygdala and 5) cerebellum. In all models and controls, high mGluR5 bindings were observed in caudate-putamen, hippocampus and lower bindings were seen in the cerebellum. Temporal changes of TACs in pilocarpine-induced epilepsy model revealed globally decreased activity in acute period compared to subacute and chronic periods after status epilepticus as well as controls. Note that standardized uptake value (SUV) is defined as the tissue concentration of [^11^C]ABP688 in the VOIs (MBq/mL) divided by the activity injected per gram of body weight (MBq/g).

### Chronic epilepsy models vs. controls

Using predefined VOIs, mGluR5 BP_ND_ of each brain region of chronic models was calculated and compared with that of controls. There was no significant difference in regional mGluR5 BP_ND_ between chronic epilepsy models and controls for the above four regions and amygdala on VOI analysis. BP_ND_ of caudate-putamen was 2.08±0.18 and 2.13±0.45, BP_ND_ of hippocampus was 1.63±0.18 and 1.52±0.43 and BP_ND_ of amygdala was 1.33±0.15 and 1.19±0.37, for controls and chronic models, respectively.

**Figure 3:**
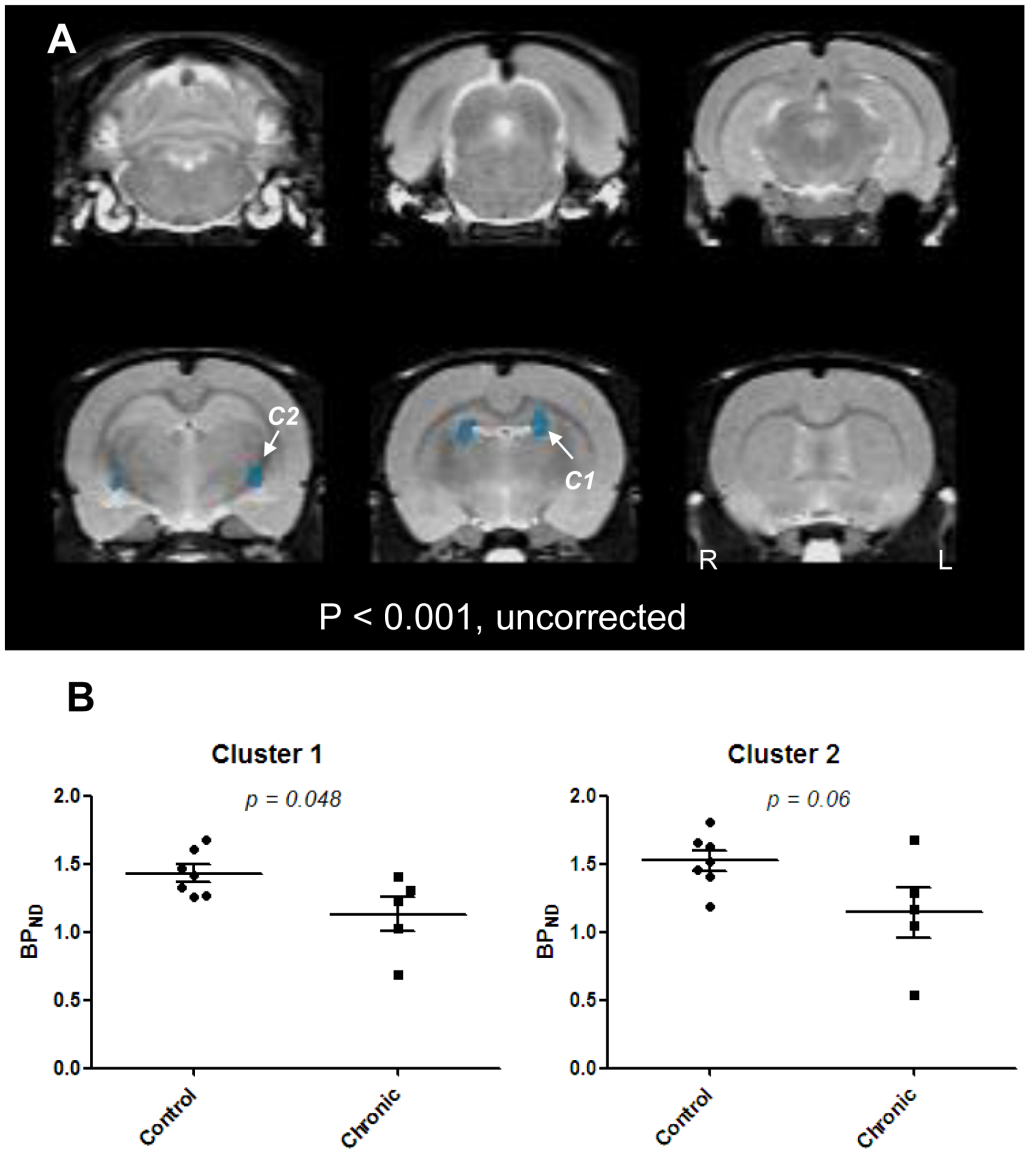
Spatial distribution of changes of mGluR5 BP_ND_ in chronic epilepsy model rats on [^11^C]ABP688 PET. Compared with controls, BP_ND_ decreased in the bilateral amygdala and dorsal hippocampi (A: p<0.001, uncorrected). (B) Nonparametric post-hoc VOI-based analysis performed on two clusters of decreased BP_ND_ on voxel-based analysis. BP_ND_ of cluster 1 (C1) on dorsal hippocampus in chronic epilepsy models decreased significantly compared to controls (p = 0.048). There was a trend for decrease in BP_ND_ of cluster 2 (C2) on amygdala in chronic epilepsy models compared to controls (p = 0.06).

Voxel-based analysis revealed the areas showing the significant difference in BP_ND_ between chronic epilepsy and controls. As is shown in Figure 3A, four clusters mainly involving the part of bilateral dorsal hippocampus and amygdala showed lower mGluR5 BP_ND_ in chronic epilepsy models than controls. Increased regional BP_ND_ was not found in the model rats.

Post-hoc analysis was performed on two clusters mainly in the left dorsal hippocampus and left amygdala among the four clusters because of relatively higher T-scores and larger cluster size (Figure 3B). BP_ND_ of the cluster 1, a VOI on dorsal hippocampus, was significantly lower in the models in chronic period than that of controls (*U* = 5, *p* = 0.048), and there was a trend for decreased BP_ND_ in chronic model in cluster 2, a VOI on amygdala (*U* = 6, *p* = 0.06).

### Temporal changes of mGluR5 BP_ND_ in acute and subacute periods

**Figure 4:**
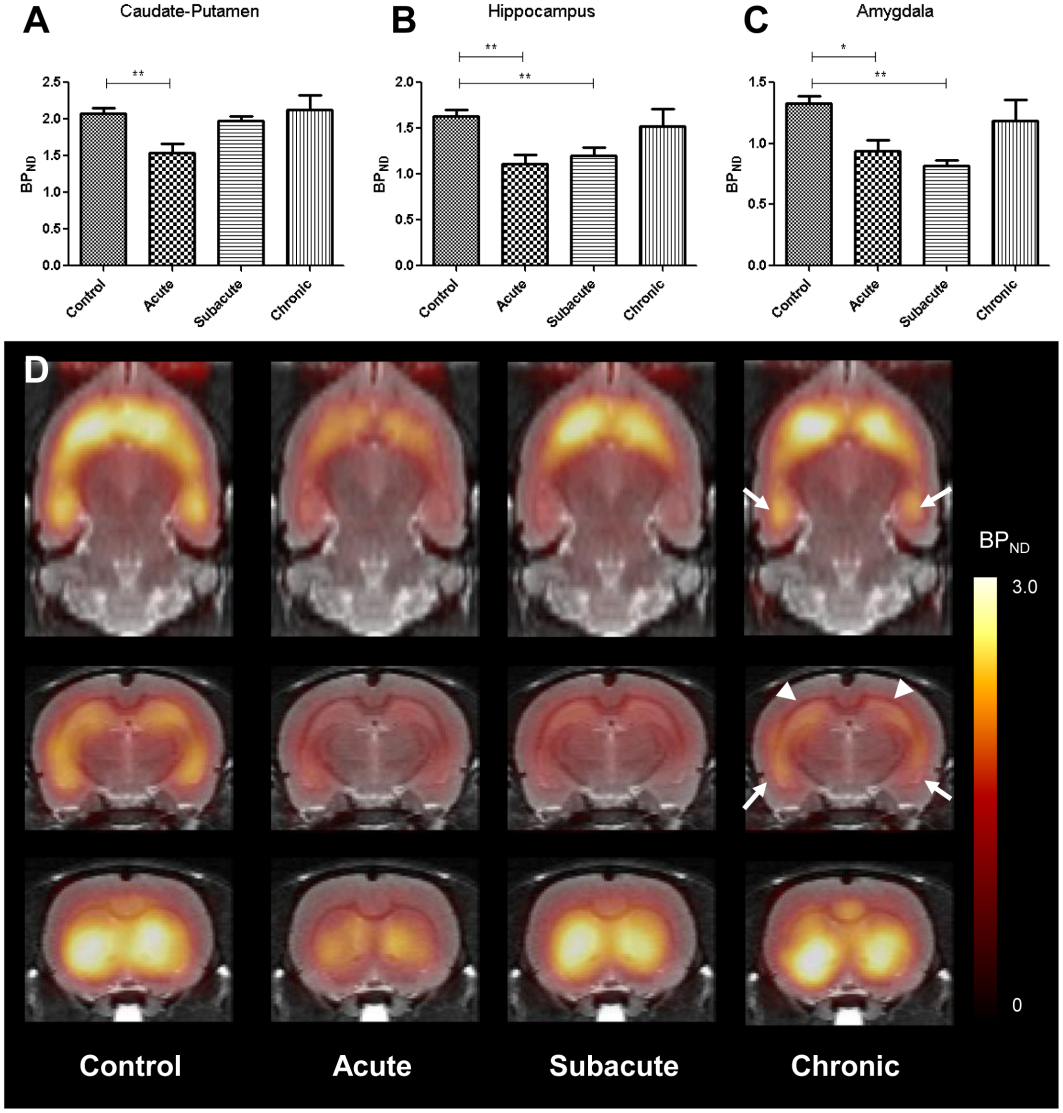
mGluR5 binding potential (BP_ND_) decreased in epilepsy models according to time periods after status epilepticus. (A–C) BP_ND_ decreased globally in acute period after status epilepticus. In subacute period, mGluR5 BP_ND_ recovered in caudate-putamen, while decreased yet in hippocampus and amygdala, and in chronic period, there was no significant difference in BP_ND_ between model rats and controls using VOI-based analysis. (D) Representative BP_ND_ parametric maps for model rats and controls were consistent with temporal patterns of VOI-based analysis. Note that BP_ND_ was visually normalized except in hippocampus and amygdala in chronic period, which corresponded to the voxel-based analysis (arrowheads: hippocampus, arrows: amygdala). Error bars represent standard errors of the mean (SEM). * p<0.05; ** p<0.01.

mGluR5 BP_ND_ of caudate-putamen decreased in acute period (1.53±0.26) compared to controls (2.13±0.45) (*U* = 0, *p*<0.01 for acute period vs. control). In the hippocampus, mGluR5 BP_ND_ of epilepsy models decreased in acute (1.11±0.20) and subacute (1.20±0.18) periods compared to controls (1.63±0.18) (*U* = 0, *p*<0.01 for acute period vs. control and for subacute period vs. control). Similarly to hippocampus, mGluR5 BP_ND_ in the amygdala was decreased in acute (0.94±0.18) and subacute (0.82±0.10) periods compared to controls (1.33±0.15) (*U* = 1, *p*<0.05 for acute period vs. control and *U* = 0, *p*<0.01 for subacute period vs. control) (Figure 4).

Parametric images of mGluR5 BP_ND_ were generated using SRTM. As shown in Figure 4D, in acute period, BP_ND_ decreased globally in the whole brain, including the caudate-putamen and hippocampus. The BP_ND_ of caudate-putamen recovered in subacute period though it was still low in hippocampus and amygdala. In chronic period, regional mGluR5 BP_ND_ in the hippocampus and amygdala VOIs (not confined to significant voxels within these regions, in that ‘significant’ means the clusters 1 or 2 in Figure 3B) recovered in comparison to that of subacute period.

## Discussion

This study analyzed mGluR5 availability of the rat brain in a pilocarpine-induced epilepsy model using [^11^C]ABP688 microPET/CT. Using SRTM, we measured mGluR5 availability by BP_ND_. In chronic epilepsy models which are characterized by spontaneous recurrent seizures, voxel-based analysis and posthoc comparison revealed that BP_ND_ in the dorsal hippocampus and amygdala was significantly lower than those of controls. We also found the temporal pattern of mGluR5 BP_ND_ change after status epilepticus, that mGluR5 BP_ND_ decreased in the rat brain of epilepsy model globally in acute period after status epilepticus. In subacute period, regional BP_ND_ in caudate-putamen was restored, though BP_ND_ in hippocampus and amygdala was still lower. Decreased mGluR5 BP_ND_ in hippocampus and amygdala sustained in chronic period in this rat model of pilocarpine-induced medial temporal lobe epilepsy.

mGluR5 expression has been studied using surgical and postmortem specimens in patients as well as in animal models [8]–[10]. However, the findings of these biopsy studies could not be obtained preoperatively. [^11^C]ABP688 PET enabled quantitative assessment of receptor availability and its serial noninvasive monitoring [13]–[15]. Reminded by the use of [^11^C]ABP688 PET to examine mGluR5 availability in patients, we observed the spatial distribution of the changes in mGluR5 availability and its temporal progress during epileptogenesis in the rat model of a chronic temporal lobe epilepsy.

In this study, the changes of mGluR5 availability were partly consistent with previous pathologic studies using *in vitro* techniques in the same pilocarpine-induced epilepsy model. In previous pathologic studies, pilocarpine-induced chronic epileptic rats revealed reduced mGluR5 expression in the hippocampus [9]. Furthermore, at 24 h after status epilepticus, mGluR5 expression decreased in amygdala, piriform and entorhinal cortices [10]. Our study revealed reduced receptor availability in bilateral hippocampi in chronic epilepsy rat models. mGluR5 receptor availability was reduced in the brain globally at 24 h after status epilepticus and this abnormality persisted in the amygdala and dorsal hippocampus in subacute and chronic periods, though the receptor availability was restored mostly in other areas in chronic period.

Pilocarpine-induced status epilepticus is characterized by generalized tonic-clonic seizures. The rats go into a seizure-free period during 2–3 weeks, followed by chronic period with spontaneous seizure activity. Decreased mGluR5 BP_ND_ in acute period after status epilepticus could be associated with postictal status, while that in subacute period might be independent to acute seizure activity because the rats were seizure-free. Thus, temporal changes in mGluR5 BP_ND_ implied gradual molecular changes during epileptogenesis rather than epileptic seizure activity itself.

In acute period after status epilepticus, overactivation of glutamatergic neuron might lead to reduced availability of mGluR5 in the entire brain. Glutamate release occupies the postsynaptic receptors after pilocarpine-induced long-lasting status epilepticus [22]. Thus, mGluR5 availability would become globally decreased in the acute period. A previous study reported increased glutamate level induced by N-acetylcystein reduced mGluR5 BP_ND_, though ABP688 is allosteric modulator [23]. In this context, excessive glutamate release during seizure could contribute to the reduction of mGluR5 BP_ND_ in acute period.

mGluR5 availability measured on [^11^C]ABP688 PET has a complex relation with mGluR5 expression of the neurons, in that various factors could contribute to the decrease of mGluR5 BP_ND_ in the epileptic rat brain. In the study by Kirschstein, et al., the decrease of mGluR5 expression was mainly caused by down-regulation of receptor molecules in the neurons but they reported that the neuronal cell loss also partially contributed to this decrease [9]. Magnetic resonance imaging (MRI) studies also showed progressively decreasing hippocampal volume in the pilocarpine-induced epilepsy model [24], [25]. Thus, both volume decrease and down-regulation should be considered when explaining the low [^11^C]ABP688 binding in the hippocampus and amygdala in the chronic period observed in our investigation. By the way, as we found that decreased BP_ND_ of mGluR5 in acute period recovered to normal already in subacute period globally in the brain, which was different from MRI findings of global progressive volume reduction [25]. In chronic period, mGluR5 BP_ND_ recovered in the entire brain except for the focal areas of bilateral amygdala and dorsal hippocampus. We speculate that down-regulation of mGluR5 receptor functional activity explains mostly the decreased BP_ND_ in hippocampus and amygdala in the chronic period of pilocarpine-induced epileptic rats. To elucidate this, further study to examine serial sequential changes of mGluR5 availability in the same individual animals during the epileptogenesis after pilocarpine-induction of epilepsy is warranted. Simultaneous [^11^C]ABP688 PET and magnetic resonance imaging as well as postmortem histologic studies might provide comprehensive epileptogenic changes in receptors and neuronal density.

One of the important issues of cross-sectional molecular studies in epilepsy is whether it underlies the epileptogenesis or result of epileptogenic processes [26]. In this study, the cause of mGluR5 BP_ND_ reduction is unknown and could be a result of epileptogenesis. One of the possible mechanisms of mGluR5 changes was an intrinsic antiepileptic response induced by status epilepticus. As mGluR5 antagonist reduces excitability, the reduction of mGluR5 availability in chronic pilocarpine-induced epilepsy may represent an endogeneous antiepileptic effort [27], [28]. This interpretation depends on the previous suggestion of feedback regulation of excitability related to neuronal homeostasis [29], [30]. Alternatively, neuronal network abnormality due to mGluR5 signal reduction would work in chronic period of pilocarpine-induced epilepsy. Decreased mGluR5 expression induced the reduction of long term depression, thus causing the abnormal activities of the neuronal network in the epileptic brain [9]. In genetic autism models, mGluR5 decrease caused network abnormalities [31], where mGluR5 abnormality underlay the pathogenesis of autistic features. Though the causal relationship between mGluR5 changes and epileptogensis is still unclear, the spatial and temporal changes in mGluR5 using [^11^C]ABP688 PET provided an essential macroscopic view of functional features of glutamatergic synapses of epileptic rat brain associated with epileptogenesis.

This study has some limitations. We studied the temporal changes in mGluR5 BP_ND_ after status epilepticus, however, the controls were only assessed at chronic periods, not at all the time points. Repeated anesthesia and imaging was difficult for seriously ill epileptic rats, though. Temporal patterns of mGluR5 BP_ND_ changes were not exactly paired and individual variation between unpaired data would have obliterated the subtle differences between groups or changes within groups. Despite this limitation, however, the results showed prominent temporal changes of BP_ND_ through the acute and subacute periods and finally in chronic period. We used ^11^C-labeled compound, thus, relatively short physical half-life compared with ^18^F-labeled compound was disadvantage in clinical application. However, compared to recently developed [^18^F]FPEB, [^11^C]ABP688 has advantages in well-established kinetics and receptor-ligand properties [15], [16]. Several mGluR5 targeted tracers including [^18^F]FPEB could be used to estimate mGluR5 availability in the future [32]. In our study, because the main purpose was to evaluate *in vivo* mGluR5 availability in epilepsy models, neither EEG monitoring of the rats nor frequency of spontaneous recurrent seizures was documented. mGluR5 imaging correlated with clinical features may provide functional grading and classification for TLE.

[^11^C]ABP688 PET promises to enable us to evaluate the mGluR5 changes during epileptogenesis and pathogenesis of neurological diseases. Because [^11^C]ABP688 PET was already used in patients with neurological and psychiatric disorders [14], [15], we propose that [^11^C]ABP688 PET be used in epilepsy patients hopefully to find seizure focus as well as to evaluate the progress of pathophysiology of epilepsy. Being reminded by the report that mGluR5 expression did not decrease but increase in the hippocampus in medial TLE patients [8], [^11^C]ABP688 PET findings should be interpreted comprehensively because they reflected the mixture of primary pathology and compensatory changes of the human brain to epileptogenesis. [^11^C]ABP688 PET might enlighten us in classifying seizure disorders in a refined fashion in terms of glutamatergic neurotransmission during pathophysiologic progress of intractable medial temporal lobe epilepsy in human as well as in rat models.

## Conclusion

We showed the *in vivo* imaging of mGluR5 in pilocarpine-induced epilepsy rat models using [^11^C]ABP688 microPET/CT. PET imaging in the present study revealed mGluR5 changes during epileptogenesis in the rat models, and also could localize abnormal mGluR5 availability associated with chronic period of epilepsy. The temporal and spatial changes of mGluR5 availability may provide abnormal glutamatergic network during epileptogenesis.

## References

[1] Ure J, Baudry M, Perassolo M (2006) Metabotropic glutamate receptors and epilepsy. J Neurol Sci 247: 1–9. 16697014 10.1016/j.jns.2006.03.018

[2] Doherty J, Dingledine R (2002) The roles of metabotropic glutamate receptors in seizures and epilepsy. Curr Drug Targets CNS Neurol Disord 1: 251–260. 12769618 10.2174/1568007023339355

[3] Bianchi R, Wong RKS, Merlin LR (2012) Glutamate Receptors in Epilepsy: Group I mGluR-Mediated Epileptogenesis. In: Noebels JL, Avoli M, Rogawski MA, Olsen RW, Delgado-Escueta AV, Jasper's Basic Mechanisms of the Epilepsies. 4th ed. Bethesda (MD). 22787676

[4] Watabe AM, Carlisle HJ, O'Dell TJ (2002) Postsynaptic induction and presynaptic expression of group 1 mGluR-dependent LTD in the hippocampal CA1 region. J Neurophysiol 87: 1395–1403. 11877514 10.1152/jn.00723.2001

[5] Merlin LR (2002) Differential roles for mGluR1 and mGluR5 in the persistent prolongation of epileptiform bursts. J Neurophysiol 87: 621–625. 11784776 10.1152/jn.00579.2001

[6] Aronica E, Gorter JA, Jansen GH, van Veelen CW, van Rijen PC, et al (2003) Expression and cell distribution of group I and group II metabotropic glutamate receptor subtypes in taylor-type focal cortical dysplasia. Epilepsia 44: 785–795. 12790891 10.1046/j.1528-1157.2003.54802.x

[7] Blumcke I, Becker AJ, Klein C, Scheiwe C, Lie AA, et al (2000) Temporal lobe epilepsy associated up-regulation of metabotropic glutamate receptors: correlated changes in mGluR1 mRNA and protein expression in experimental animals and human patients. J Neuropathol Exp Neurol 59: 1–10. 10744030 10.1093/jnen/59.1.1

[8] Notenboom RG, Hampson DR, Jansen GH, van Rijen PC, van Veelen CW, et al (2006) Up-regulation of hippocampal metabotropic glutamate receptor 5 in temporal lobe epilepsy patients. Brain 129: 96–107. 16311265 10.1093/brain/awh673

[9] Kirschstein T, Bauer M, Muller L, Ruschenschmidt C, Reitze M, et al (2007) Loss of metabotropic glutamate receptor-dependent long-term depression via downregulation of mGluR5 after status epilepticus. J Neurosci 27: 7696–7704. 17634364 10.1523/JNEUROSCI.4572-06.2007 PMC6672893

[10] Cavarsan CF, Tescarollo F, Tesone-Coelho C, Morais RL, Motta FL, et al (2012) Pilocarpine-induced status epilepticus increases Homer1a and changes mGluR5 expression. Epilepsy Res 101: 253–260. 22591751 10.1016/j.eplepsyres.2012.04.011

[11] Enz R (2012) Metabotropic glutamate receptors and interacting proteins: evolving drug targets. Curr Drug Targets 13: 145–156. 21777188 10.2174/138945012798868452

[12] Ametamey SM, Kessler LJ, Honer M, Wyss MT, Buck A, et al (2006) Radiosynthesis and preclinical evaluation of ^11^C-ABP688 as a probe for imaging the metabotropic glutamate receptor subtype 5. J Nucl Med 47: 698–705. 16595505

[13] Deschwanden A, Karolewicz B, Feyissa AM, Treyer V, Ametamey SM, et al (2011) Reduced metabotropic glutamate receptor 5 density in major depression determined by [^11^C]ABP688 PET and postmortem study. Am J Psychiatry 168: 727–734. 21498461 10.1176/appi.ajp.2011.09111607 PMC3129412

[14] Akkus F, Ametamey SM, Treyer V, Burger C, Johayem A, et al (2013) Marked global reduction in mGluR5 receptor binding in smokers and ex-smokers determined by [^11^C]ABP688 positron emission tomography. Proc Natl Acad Sci U S A 110: 737–742. 23248277 10.1073/pnas.1210984110 PMC3545768

[15] Ametamey SM, Treyer V, Streffer J, Wyss MT, Schmidt M, et al (2007) Human PET studies of metabotropic glutamate receptor subtype 5 with ^11^C-ABP688. J Nucl Med 48: 247–252. 17268022

[16] Elmenhorst D, Minuzzi L, Aliaga A, Rowley J, Massarweh G, et al (2010) In vivo and in vitro validation of reference tissue models for the mGluR(5) ligand [^11^C]ABP688. J Cereb Blood Flow Metab 30: 1538–1549. 20531460 10.1038/jcbfm.2010.65 PMC2949244

[17] Racine R, Okujava V, Chipashvili S (1972) Modification of seizure activity by electrical stimulation. 3. Mechanisms. Electroencephalogr Clin Neurophysiol 32: 295–299. 4110398 10.1016/0013-4694(72)90178-2

[18] Lee HJ, Jeong JM, Lee YS, Kim HW, J.Y C, et al (2009) A convenient radiolabeling of [^11^C](R)-PK11195 using loop method in automatic synthesis module. Nucl Med Mol Imaging 43: 337–343.

[19] Schiffer WK, Mirrione MM, Biegon A, Alexoff DL, Patel V, et al (2006) Serial microPET measures of the metabolic reaction to a microdialysis probe implant. J Neurosci Methods 155: 272–284. 16519945 10.1016/j.jneumeth.2006.01.027

[20] Innis RB, Cunningham VJ, Delforge J, Fujita M, Gjedde A, et al (2007) Consensus nomenclature for in vivo imaging of reversibly binding radioligands. J Cereb Blood Flow Metab 27: 1533–1539. 17519979 10.1038/sj.jcbfm.9600493

[21] Lammertsma AA, Hume SP (1996) Simplified reference tissue model for PET receptor studies. Neuroimage 4: 153–158. 9345505 10.1006/nimg.1996.0066

[22] Costa MS, Rocha JB, Perosa SR, Cavalheiro EA, Naffah-Mazzacoratti Mda G (2004) Pilocarpine-induced status epilepticus increases glutamate release in rat hippocampal synaptosomes. Neurosci Lett 356: 41–44. 14746897 10.1016/j.neulet.2003.11.019

[23] Miyake N, Skinbjerg M, Easwaramoorthy B, Kumar D, Girgis RR, et al (2011) Imaging changes in glutamate transmission in vivo with the metabotropic glutamate receptor 5 tracer [^11^C] ABP688 and N-acetylcysteine challenge. Biol Psychiatry 69: 822–824. 21288506 10.1016/j.biopsych.2010.12.023

[24] Niessen HG, Angenstein F, Vielhaber S, Frisch C, Kudin A, et al (2005) Volumetric magnetic resonance imaging of functionally relevant structural alterations in chronic epilepsy after pilocarpine-induced status epilepticus in rats. Epilepsia 46: 1021–1026. 16026554 10.1111/j.1528-1167.2005.60704.x

[25] Nairismagi J, Pitkanen A, Kettunen MI, Kauppinen RA, Kubova H (2006) Status epilepticus in 12-day-old rats leads to temporal lobe neurodegeneration and volume reduction: a histologic and MRI study. Epilepsia 47: 479–488. 16529609 10.1111/j.1528-1167.2006.00455.x

[26] Merlin LR (2008) The ups and downs of hippocampal metabotropic glutamate receptors: ramifications for epileptogenesis and cognitive impairment following status epilepticus. Epilepsy Curr 8: 43–45. 18330467 10.1111/j.1535-7511.2008.00232.x PMC2265804

[27] Tang FR, Chen PM, Tang YC, Tsai MC, Lee WL (2007) Two-methyl-6-phenylethynyl-pyridine (MPEP), a metabotropic glutamate receptor 5 antagonist, with low doses of MK801 and diazepam: a novel approach for controlling status epilepticus. Neuropharmacology 53: 821–831. 17904168 10.1016/j.neuropharm.2007.08.012

[28] Lojkova-Janeckova D, Ng J, Mares P (2009) Antagonists of group I metabotropic glutamate receptors and cortical afterdischarges in immature rats. Epilepsia 50: 2123–2129. 19486355 10.1111/j.1528-1167.2009.02091.x

[29] Ramocki MB, Zoghbi HY (2008) Failure of neuronal homeostasis results in common neuropsychiatric phenotypes. Nature 455: 912–918. 18923513 10.1038/nature07457 PMC2696622

[30] Sakagami Y, Yamamoto K, Sugiura S, Inokuchi K, Hayashi T, et al (2005) Essential roles of Homer-1a in homeostatic regulation of pyramidal cell excitability: a possible link to clinical benefits of electroconvulsive shock. Eur J Neurosci 21: 3229–3239. 16026461 10.1111/j.1460-9568.2005.04165.x

[31] Auerbach BD, Osterweil EK, Bear MF (2011) Mutations causing syndromic autism define an axis of synaptic pathophysiology. Nature 480: 63–68. 22113615 10.1038/nature10658 PMC3228874

[32] Wang JQ, Tueckmantel W, Zhu A, Pellegrino D, Brownell AL (2007) Synthesis and preliminary biological evaluation of 3-[^18^F]fluoro-5-(2-pyridinylethynyl)benzonitrile as a PET radiotracer for imaging metabotropic glutamate receptor subtype 5. Synapse 61: 951–61. 17787003 10.1002/syn.20445

